# Predictive Value of Pin1 in Cervical Low-Grade Squamous Intraepithelial Lesions and Inhibition of Pin1 Exerts Potent Anticancer Activity against Human Cervical Cancer

**DOI:** 10.14336/AD.2019.0415

**Published:** 2020-02-01

**Authors:** Yan-Tong Guo, Yan Lu, Yi-Yang Jia, Hui-Nan Qu, Da Qi, Xin-Qi Wang, Pei-Ye Song, Xiang-Shu Jin, Wen-Hong Xu, Yuan Dong, Ying-Ying Liang, Cheng-Shi Quan

**Affiliations:** Key Laboratory of Pathobiology, Ministry of Education, College of Basic Medical Sciences, Jilin University, Changchun, China.

**Keywords:** Pin1, SIL, cell death, EMT, C-Jun, KPT-6566, cervical cancer

## Abstract

Many oncogenes are involved in the progression from low-grade squamous intraepithelial lesions (LSILs) to high-grade squamous intraepithelial lesions (HSILs); which greatly increases the risk of cervical cancer (CC). Thus, a reliable biomarker for risk classification of LSILs is urgently needed. The prolyl isomerase Pin1 is overexpressed in many cancers and contributes significantly to tumour initiation and progression. Therefore, it is important to assess the effects of cancer therapies that target Pin1. In our study, we demonstrated that Pin1 may serve as a biomarker for LSIL disease progression and may constitute a novel therapeutic target for CC. We used a the novel Pin1 inhibitor KPT-6566, which is able to covalently bind to Pin1 and selectively target it for degradation. The results of our investigation revealed that the downregulation of Pin1 by shRNA or KPT-6566 inhibited the growth of human cervical cancer cells (CCCs). We also discovered that the use of KPT-6566 is a novel approach to enhance the therapeutic efficacy of cisplatin (DDP) against CCCs in vitro and in vivo. We showed that KPT-6566-mediated inhibition of Pin1 blocked multiple cancer-driving pathways simultaneously in CCCs. Furthermore, targeted Pin1 treatment suppressed the metastasis and invasion of human CCCs, and downregulation of Pin1 reversed the epithelial-mesenchymal transition (EMT) of CCCs via the c-Jun/slug pathway. Collectively, we showed that Pin1 may be a marker for the risk of progression to HSIL and that inhibition of Pin1 has anticancer effects against CC.

Cervical cancer is one of the most common malignant tumours and the second most common cause of cancer-related deaths in women worldwide. Importantly, the continued development of pathology technologies has allowed for CC to be detected in squamous intraepithelial lesions (SILs) [1]. However, there are no reliable indicators to better risk-stratify LSIL cases and to prevent the overtreatment or neglect treatment of CC [2].

At present, surgery and radiotherapy are the primary treatments for early cervical cancer, but the current treatment regimens are known to have shown limited survival benefits for advanced cancer stages and in reoccurring cases [3]. The infiltration, migration, invasion and the chemoresistance of CCCs are the primary reasons for the low five-year survival rate of CC patients [4]. The most common treatments include cisplatin-based chemoradiation, and the main challenge of this therapy is in improving its efficacy [5]. Due to the activation and feedback of multiple cancer-driving pathways, about 30% of patients experience lymph node recurrence and distant metastasis after cisplatin-based treatment. Therefore, this treatment fails to fully eradicate the disease [6]. Many kinases and phosphatases are involved in the associated signalling mechanisms [7]. A unique enzyme, Pin1, mediates the dynamic crosstalk between multiple molecular pathways and participates in various stages of tumourigenesis [8].

Pin1 is a member of the peptidylprolyl isomerase (PPIase) family that has been reported to be overexpressed in many cancers and is associated with a poor prognosis [9]. Some proteins that contain phosphorylated Ser/Thr-Pro motifs, such as c-Jun, β-catenin and NF-κB, are specific target substrates of Pin1 [10]. The prolyl isomerase Pin1 is a pivotal catalyst for tumour progression that involves proline-directed phosphorylation [11]. Pin1 promotes tumourigenesis by disrupting the balance between oncogenes and tumour suppressors, and activates many cancer-driving pathways [12]. Genetic knockdown of Pin1 reduces tumour growth and metastasis in many cancers, such as breast, lung and liver cancer [13-15]. In addition, the suppression of Pin1 leads to the sensitization of breast cancer cells to different drugs [16]. Previous work performed by our lab has shown that the expression of Pin1 in the CRL cell line, which is resistant to Herceptin, was significantly higher than that of BT-474 in triple positive breast cancer cell lines (data not shown). Altogether, these data strongly provide a compelling rationale for the study research of Pin1-targeted therapies.

The activation of oncogenes and the low expression levels of Pin1 in normal tissues make Pin1 an attractive target for anticancer drugs [17]. Although many inhibitors, including juglone, PiB and buparvaquone, have been isolated thus far, their unsatisfactory pharmacological performance with respect to potency, specificity, solubility, cell permeability and stability has limited their clinical trial phase progression [9, 18]. KPT-6566 is a novel Pin1-specific inhibitor that was discovered and synthesized by Elena Campaner* et al*. Furthermore, KPT-6566 covalently binds to the catalytic site of it and targets Pin1 for degradation. KPT-6566 is able to specifically inhibit the vitality of Pin1-overexpressing cancer cells while not affecting normal cells [19]. Thus, KPT-6566 has significant therapeutic effects on CCCs in vitro and in vivo, and it is necessary to elucidate its associated molecular mechanisms.

In this study, the results of our clinical data analysis demonstrated that Pin1 was significantly associated with the poor outcomes of LSIL patients. The inhibition of Pin1 expression by gene knockout or KPT-6566 significantly inhibited CCC invasion and metastasis and significantly enhanced the killing effect of cisplatin on CCCs in vitro and in vivo. Moreover, the treatment of KPT-6566 had no obvious side effects on nude mice, and we also demonstrated that it is highly compatible with cisplatin. These results reveal that Pin1 may be a marker for risk of progression of LSIL to a high-grade lesion of LSIL and a promising therapeutic target for CC.

## MATERIALS AND METHODS

### Cell line cultures

The cell lines HeLa and SiHa were purchased from the Cell Bank of the Chinese Academy of Sciences. The cells were maintained in HDMEM (#0014316; Kibbutz Beit Haemek, Israel) supplemented with 10% foetal bovine serum (#1706126; Kibbutz Beit Haemek, Israel) and 1.5 g/L of NaHCO_3_. All cell lines were cultured in a humidified atmosphere with 5% CO_2_ at 37 °C.

### Cell transfection

A Pin1 shRNA plasmid, i.e., PIN1-RNA (#3763-1; GENECHEM, Shanghai, China), was constructed, that also expresses green fluorescent protein (GFP). The target sequence was GATTTGAAGAAGACGCCTCGTT, and a functional non-targeting siRNA was used as a control. The c-Jun overexpression plasmid, i.e., pECMV-3*FLAG-JUN (#1801293; MiaoLingBio, Changchun, China), was transfected into HeLa-shPin1/SiHa-shPin1 cells, and a functional, non-targeting siRNA was used as a control.

### Western blotting analysis

The total protein content of the cells was extracted with RIPA lysis buffer (Dingguo, Beijing, China) supplemented with protease inhibitor (Roche, Basel, Switzerland). Subsequently, the proteins were loaded and separated via 10-12% sodium dodecyl sulphate-polyacrylamide gel electrophoresis, after which the proteins were transferred to 0.22-μm polyvinylidene fluoridemembranes (Millipore, MA, USA). The membranes were blocked with 5% non-fat milk (BD Biosciences, CA, USA) and were sequentially incubated with the primary and secondary antibodies.

The protein samples were incubated with the following antibodies: rabbit anti-cyclin D1 (#2978 S; Cell Signaling Technology, MA, USA) 1:1000, rabbit anti-Pin1 (#ab191271; Abcam, Cambridge, England) 1:700, rabbit anti-E-cadherin (#3195 T; Cell Signaling Technology, MA, USA) 1:1000, rabbit anti-N-cadherin (#13116 T; Cell Signaling Technology, MA, USA) 1:1000, rabbit anti-vimentin (#5741 T; Cell Signaling Technology, MA, USA) 1:1000, rabbit anti-c-Jun (#9165 S; Cell Signaling Technology, MA, USA) 1:1000, rabbit anti-slug (#9585 T; Cell Signaling Technology, MA, USA) 1:1000, rabbit anti-cleaved-caspase3 (#9665 S; Cell Signaling Technology, MA, USA) 1:1000, rabbit anti-cleaved-PARP (#9532 T; Cell Signaling Technology, MA, USA) 1:1000, rabbit anti-β-catenin (#8480 T; Cell Signaling Technology, MA, USA) 1:1000, rabbit anti-H2A.X (#7631 T; Cell Signaling Technology, MA, USA) 1:1000, rabbit anti-p65/NF-κB (#8242 T; Cell Signaling Technology, MA, USA) 1:1000, mouse anti-GSTP1 (#3369 S; Cell Signaling Technology, MA, USA) 1:1000, and mouse anti-Actin (#HC201; Proteintech, Wuhan, China) 1:5000. Finally, the blots were visualized using an electrochemiluminescence system (TECAN, Beijing, China).

The normal cervical and cervical cancer tissues assayed by western blotting were collected from the human biological library of the Second Hospital of Jilin University. We randomly took three samples from each group and extracted proteins for western blotting. β-actin was used as an endogenous control.

### Patients and tissue specimens

A total of 110 formalin-fixed, paraffin-embedded cervical tissue specimens were analysed, with the samples were obtained from the archived files of patients treated at the Department of Pathology at the Second Hospital of Jilin University between 2009 and 2010. Twenty-eight cases were squamous cervical cancer (SCC), 10 were HSIL, and 72 were LSIL. All LSIL cervical biopsy patients had received follow-up pathological examinations (either tissue or cytology examinations) through December 2018, i.e., for approximately 7-9 years. The diagnostic endpoint was determined by the highest-grade histological finding. The cases were classified into the two following groups: in the first group, the diagnostic endpoints were LSIL, including atypical or benign cases that corresponded to the persistence of a low-grade lesion, a new LSIL, or regression; in the second group, the diagnostic endpoint was progression to HSIL, which is defined as the presence of at least cervical intraepithelial neoplasia (CIN) greater than a grade of 2. The clinicopathological data of the SCC patients were retrieved from the patients’ medical records. These parameters included the patient’s age at initial diagnosis, tumour size and lymph node metastasis.

### Immunohistochemical staining

Patient cervical tissues (n = 110) including SCC and SIL tissues were retrieved from The Pathology Department at the Second Hospital of Jilin University for immune-histochemistry staining. The latter tissues were used to re-confirm the diagnoses. The tumour histological grades were assessed using the Nottingham grading system. The clinicopathological data included the patient’s age, initial diagnosis, histological subtype, tumour size, lymph node metastasis from the patient’s medical records. The paraffin sections were cut to a thickness of 2 μm, deparaffinized in xylene, rehydrated in a series of ethanol solutions (100 to 50%), and incubated in a 0.3% hydrogen peroxide solution for 20 minutes to block endogenous peroxidase activity. The tissue sections were subsequently rinsed with tap water, washed with phosphate-buffered saline (PBS) and subjected to antigen retrieval by boiling in a Tris/EDTA (pH 9.0) solution for 20 minutes. Subsequently, the sections were cooled, rinsed with tap water and PBS, and incubated with normal serum at room temperature for 30 minutes; Subsequently, the samples were incubated overnight incubation with a primary rabbit antibody against Pin1 (#191271; Abcam, MA, USA; 1:200 dilution in PBS), c-Jun (#9165S; Cell Signaling Technology, MA, USA; 1:400 dilution in PBS), Ki67 (#27309-1-AP, Porteintech, Wuhan, China; 1:400 dilution in PBS) and p16 (#ZM-0205, Zhongshan Golden Bridge, Beijing, China; 1:200 dilution in PBS) at 4 °C. The next day, the sections were washed three times with PBS and then incubated with a secondary antibody using KIT-9270 IHC Kit (MXB Biotechnology, Fuzhou, China) at room temperature for 30 minutes. The colour reaction was developed using a 3’-diaminobenzidine (DAB) kit (Zhongshan Golden Bridge Biotechnology, Beijing, China). The immunostained tissue sections were independently reviewed and scored by two investigators to determine the percentages of immunostaining and the staining intensity, as described previously. A numerical final Pin1/c-Jun expression score (FS = P × I) was calculated for each tissue by multiplying the staining intensity (I) score (0, none; 1, weak; 2, medium; 3, strong) by the percentage (P) of positively stained cells (0-100). The FS ranged from 0 to 300. Next, each case was scored as positive or negative using the median FS as the cut-off value for the log-rank test, which is the most unbiased, albeit stringent, criterion for data analysis according to a previous study.

### RNA extraction and quantitative reverse transcription PCR

The total RNA was isolated from 1 × 10^7^ cells using TRIzol Reagent (Invitrogen, CA, USA). The cDNA was synthesized from the RNA using an RT-PCR reverse transcription kit (TransGen Biotech, Beijing, China). Two micrograms of total RNA were reverse transcribed into cDNA under at: 25 °C for 10 minutes, 42 °C for 30 minutes, and 85 °C for 5s, according to the manufacturer’s specifications. Subsequently, the cDNA was stored at -20 °C until use. PCR was performed using a PCR kit (TransGen Biotech, Beijing, China), using primers that were synthesized by Sangon (Sangon, Shanghai, China). Quantitative PCR was carried out with either Taq-Man or SYBR Green PCR reagents using an ABI Prism 7300 detection system (all from Applied Biosystems, Foster City, CA). The reaction programme consisted of 95 °C for 3 minutes, followed by 40 cycles of 95 °C for 30 s, 55 °C for 20 s, and 72 °C for 15 s. The GAPDH gene served as an internal control, and the relative mRNA levels were calculated by 2^-ΔΔCt^. The experiments were repeated in triplicate.

### CCK8 assay

The in vitro drug cytotoxicity was measured using the Cell Counting Kit-8 (CCK-8) assay (Dojindo, Kumamoto, Japan). The cells were seeded into 96-well plates (2 × 10^3^ cells/well) and were then treated for 48 h in 100 μl of medium with the appropriate drug. The cells that were incubated without drugs (i.e., the control wells) were set at a 100% survival rate and were utilized to calculate the concentration of each cytostatic drug that was lethal to 50% of the cells (IC50). Ten microliters of CCK8 was added to 90 µl of media, after which the cells were then incubated at 37 °C with 5% CO_2_ for one hour. The absorbance was recorded at a wavelength of 450 nm using a microplate reader (Thermo, Schwerte, Germany), and the assays were repeated in triplicate.

### FACS assay

The cells (1×10^6^) were harvested using trypsin (without EDTA) and were separated into two groups. Next the cells were washed with cold PBS; then, the cells were centrifuged, and the supernatant was discarded. Subsequently, an Annexin V-APC Apoptosis Analysis kit (Sungene Biotech, Tianjin, China) were used to detect cell apoptosis. The first tube was a blank control, the second tube contained 5 μl of Annexin V-APC, and the third tube contained 5 μl of the 7AAD solution. Each tube was gently vortexed and incubated for 5 minutes at room temperature, protected from light. Before an analysis by flow cytometry analysis, the blank control and single dye samples were used to regulate the voltage and compensation.

### Animal experiments

Female BALB/c nude mice (16-18 g) were purchased from the Beijing Laboratory Animal Center (Beijing, China). The mice were maintained in the Animal Experiment Center at the Basic Medical College of Jilin University and were injected subcutaneously with 1 × 10^7^ tumour cells in 500 μl PBS medium, with the tumours collected after 8 weeks.The tumours were measured using a Vernier calliper, and the volume was calculated using the following formula: V = (width^2^ × length)/2.

For liver metastasis assays in vivo, nude mice were randomly separated into four groups (n = 3) and were subjected to laparotomy after anaesthesia with isoflurane. For each mouse, 1 × 10^6^ cells in 100 μl PBS was injected into the distal tip of the spleen with an insulin syringe. Ten weeks after the injections, the mice were euthanized, and the livers were collected for staining. The mouse liver sections were stained with haematoxylin and eosin (H&E), and the metastatic nodules and necrotic areas were counted manually counted using a microscope.

### Cell migration and invasion assay

The cells (1 × 10^6^) were seeded into 6-well plates, and a sterile 100-μl pipette tip was used to scratch 3 lines when the cells reached 95% confluence. The cells were cultured with fresh medium for 24 h and then photographed to calculate the wound closure using a microscope.

Transwell assays were performed to evaluate the cell invasion. The transwell assays were performed using chambers containing Matrigel (8-μm pore; Corning, USA). For this assay, 1 × 10^6^ cells in serum-free medium were seeded into the cell culture inserts, after which complete medium supplemented with 10% FBS (Biological Industries, Kibbutz Beit Haemek, Israel) was added to the bottom chamber. After incubating for 24 h, the cells invaded the lower surface of the insert filter. Subsequently, the cells were fixed with 4% paraformaldehyde (DingGuo, Beijing, China), stained with crystal violet and counted using a microscope.

### TUNEL assay

For the TUNEL assay, after exposure to celastrol for 24 h, the tissue sections were fixed with 4% paraformaldehyde. TUNEL assays were performed according to the manufacturer’s protocol (Roche, Basel, Switzerland). Subsequently, the cells were then stained with DAPI (Invitrogen, CA, USA) and were observed by fluorescence microscopy.

### Statistical analysis

The data are expressed as the means ± standard deviation and were analysed using an unpaired Student’s t-test. P < 0.05 was considered to be significant. All graphs and statistical calculations were performed using GraphPad Prism (Version 6.0; La Jolla, CA, USA).

**Figure 1.**
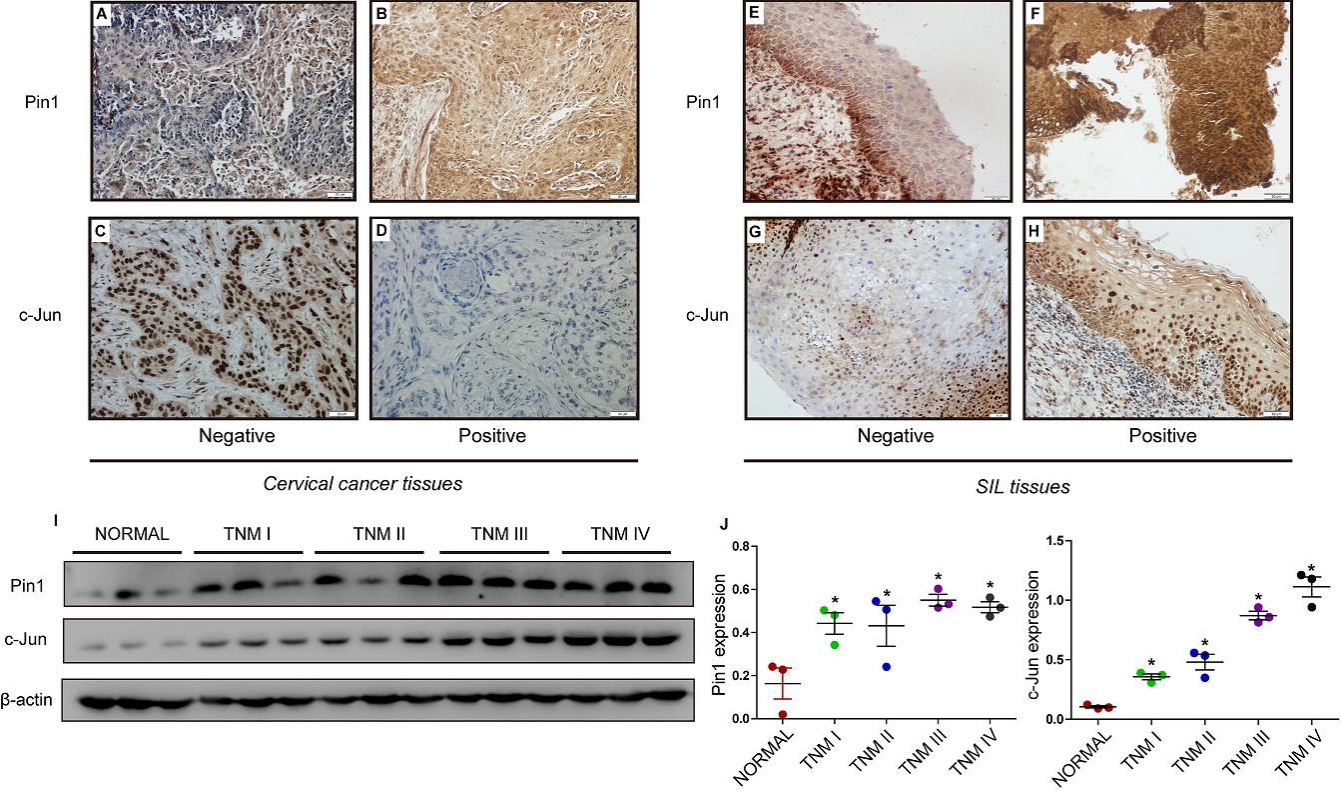
**The expression of Pin1 and c-Jun in SILs and cervical cancer patient tissues**. Prognostic value of Pin1 in LSIL patients. **(A-D)** Low vs. high expression of Pin1/c-Jun in cervical cancer issue specimens; (E-H) Low vs. high expression of Pin1/c-Jun in squamous intraepithelial lesions specimens. (original magnification × 200). (I, J) The expression of Pin1 and c-Jun in normal cervical tissues and different stages of cervical cancer patients detected by western blot. (* compared with normal group, P<0.05).

## RESULTS

### Pin1 and c-Jun in SILs and cervical cancer patient tissues; Pin1 positivity was significantly associated with higher HSIL progression rates in LSIL patients

To investigate the roles of Pin1 and c-Jun in cervical cancer, we first examined the expression of Pin1/c-Jun in LSILs, HSILs and SCC patient tissues. IHC was performed using paraffin-embedded tissue sections (n = 110) of histopathologically confirmed SCC (Fig. 1A-D) and SIL (Fig. 1E-G). The progression of LSIL to HSIL greatly increases the risk of cervical cancer. After a follow-up of 72 LSIL patients, 3 of 37 (8%) were observed to have HSIL progression in Pin1-negative LSIL cases, while 14 of 35 (40%) were observed to have HSIL progression in Pin1-positive LSIL cases. There was no significant difference in the progression rate (LSIL to HSIL) in c-Jun-negative LSIL cases (20%) and c-Jun-positive LSIL cases (25%). Pin1 expression was correlated with poor outcomes for LSIL patients (P= 0. 001), whereas c-Jun expression was irrelevant to the outcomes of LSIL patients (P= 0.599) (Table 1).

**Table 1:** Pin1 and c-Jun statuses in patients on follow-up examination

	Characteristics		No.	Outcome		*P* value
				LSIL	HSIL	
LSIL	Pin1 status	Negative	37	34	3	0.001
		Positive	35	21	14	
	c-Jun status	Negative	25	20	5	0.599
		Positive	47	35	12	

Next, we investigated the relationship between Pin1/c-Jun expression and patient characteristics. The proportion of Pin1 detected in SCC cases with or without lymph node metastasis was 100 % and 54.5 %, respectively. We concluded that Pin1 expression was positively associated with lymph node metastasis (P = 0.039) and Ki67 (P=0.014) but was not correlated with patient age (P = 0.724), tumour size (P = 0.927) and P16 (P=NA). C-Jun expression was irrelevant to patient age (P = 0.662), lymph node metastasis (P = 0.443), tumour size (P = 0.611) or P16 (P=NA) but was associated with Ki67 (P<0.001) (Table 2). C-Jun was detected in 48.6% of Pin1-negative LSIL cases, 82.6% of Pin1-positive LSIL cases, 80% of Pin1-negative SCC cases, and 100% of Pin1-positive SCC cases. Pin1 expression was positively associated with c-Jun expression in LSIL cases (P = 0.002) and SCC cases (P = 0.049) but was not correlated with c-Jun expression in HSIL cases (P = 0.747) (Table 3). Positive Pin1 expression was observed in 48.6% (35/72) of LSIL tissues, 60% (6/10) of HSIL tissues, and 64.3% (18/28) of SCCs. The latter rate was higher than that observed for HSIL and LSIL, although the trend was not significant (P = 0.338). C-Jun expression was significantly different among the different groups (P = 0.017), with the highest expression observed in SCCs and the lowest in LSIL.

To explore the role of Pin1 and c-Jun in cervical cancer progression, we assessed the expression of Pin1 and c-Jun in normal cervical tissues and cancerous cervical tissues by western bloting. The results showed that Pin1 and c-Jun expression was significantly higher in cervical cancer tissue than that in normal cervical tissue. Furthermore, the protein levels of Pin1 and c-Jun were significantly increased in high grade cervical cancer (TNM III, TNM IV) (Fig. 1I, J), demonstrating that increased levels of Pin1 and c-Jun were associated with the TNM stage of human cervical cancer.

**Table 2:** Pin1 and c-Jun statuses according to Age, Lymph node status, Tumor size, P16 and Ki67.

Characteristics		No.	Pin1 status		*P* value	C-jun statuses		*P* value
			*Negative No.* (%)	*Positive No.* (%)		*Negative No.* (%)	*Positive No.* (%)	
Age (years)	<50	10	4	6	0.724	1	9	0.662
	≥50	18	6	12		1	17	
Lymph node status	Absent	22	10	12	**0.039**	2	20	0.443
	Present	6	0	6		0	6	
Tumor size	≤4 cm	25	9	16	0.927	2	23	0.611
	>4 cm	3	1	2		0	3	
p16	Negative	0	0	0	NA	0	0	NA
	Positive	28	10	18		2	26	
Ki67	1%-25%	0	0	0	**0.014**	0	0	**<0.001**
	25%-50%	0	0	0		0	0	
	50%-75%	3	3	0		2	1	
	>75%	25	7	18		0	25	

### Inhibition of Pin1 suppressed the cell proliferation of CCCs

**Figure 2.**
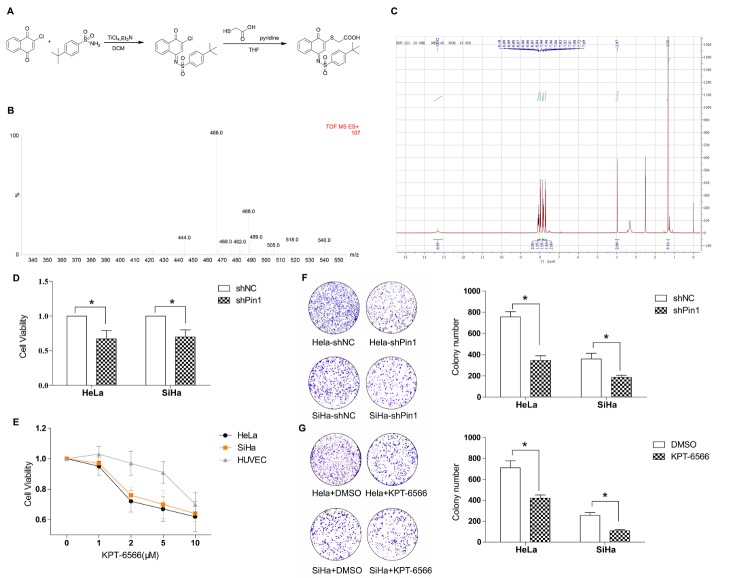
**Genic or chemical downregulation of Pin1 suppressed cell proliferation in CCCs**. **(A)** Chemical synthesis steps of KPT-6566. **(B)** Mass spectrum of KPT-6566, ESI-MS: m/z 466.0 [M^+^Na]^+^. **(C)** Hydrogen spectroscopy to confirm chemical structure of KPT-6566, 1H NMR (300 MHz,DMSO-d6) δ 8.14 - 8.04 (m, 2H), 8.03 -7.97 (m, 2H), 7.90 - 7.87 (s, 1H), 7.87 - 7.80 (m, 2H), 7.76 - 7.69 (m, 2H), 3.99 (s, 2H), 1.35 (s, 9H). **(D)** Cell viability assay of the Hela-shPin1/Hela-shNC and SiHa-shPin1/SiHa-shNC for 24 h. **(E)** Hela, SiHa or HUVEC cells were treated with KPT-6566 and the growth curves were plotted over concentration. **(F)** Representative micrographs of the colonies of Hela-shPin1/SiHa-shPin1 were counted and compared with that of NC. **(G)** Representative micrographs of the colonies of Hela/SiHa treated with KPT-6566 were counted and compared with that of treated with DMSO. Each assay was performed in triplicate. *P<0.05.

We previously showed that Pin1 expression is a key event in clinical cervical cancer tissue cases, but the therapeutic potential of Pin1 in treating CC is still unclear. We first established the SiHa stable cell line SiHa-shPin1 and the HeLa stable cell line HeLa-shPin1 to suppress Pin1 expression as well as the control stable cell lines SiHa-shNC and HeLa-shNC. Both the mRNA and protein levels of Pin1 were confirmed by qPCR and western blotting. We next synthesized the novel Pin1-specific inhibitor KPT-6566 as follows (Fig. 2A): Compound B (326 mg, 2 mmol) was dissolved in dichloromethane (15 ml), compound A (385 mg, 2 mmol) was added, the reaction mixture was brought to 0 °C, and 1 mol of titanium tetrachloride in dichloromethane was slowly added dropwise. The solution (2 ml) and triethylamine (0.613 ml, 4.4 mmol) were reacted and warmed to 60 °C. When the reaction did not occur, the reaction solution was cooled to room temperature, the solvent was evaporated, and the residue was dissolved in 100ml of ethyl acetate. The insoluble materials were removed by filtration, the filtrate was concentrated and purified by silica gel column chromatography (petroleum ether: ethyl acetate = 20:1), to yield compound C (yellow solid, 435 mg, yield 56%). Compounds C (200 mg, 0.5 mmol) and D (0.036 ml, 0.5 mmol) were dissolved in EtOAc after the TLC reaction was completed. The insoluble material was removed by filtration, and the filtrate was concentrated and purified by silica gel column chromatography (ethyl ether: ethyl acetate = 1:1) to yield compound E (yellow solid, 140 mg, yield 61%). To verify our synthesis results, we performed mass spectrometry (Fig. 2B) and hydrogen spectroscopy (Fig. 2C) to confirm the chemical structures.

Next, we examined the effect of the loss of Pin1 on the functions of the HeLa and SiHa cell lines. The CCK8 assay results showed that the cell viability of the HeLa/SiHa cells was significantly reduced. HeLa/SiHa cell growth was significantly impaired (Fig. 2D) as demonstrated by obtained growth curves that were based on the drug concentrations compared to the cell growth of HUVECs (Fig. 2E). HeLa/SiHa colony formation was significantly decreased after the suppression of Pin1 by shRNA or KPT-6566. We observed that the lack of Pin1 had a great effect on human CCCs (Fig. 2F, G). These results indicated that the proliferation of human CCCs is likely associated with Pin1.

**Figure 3.**
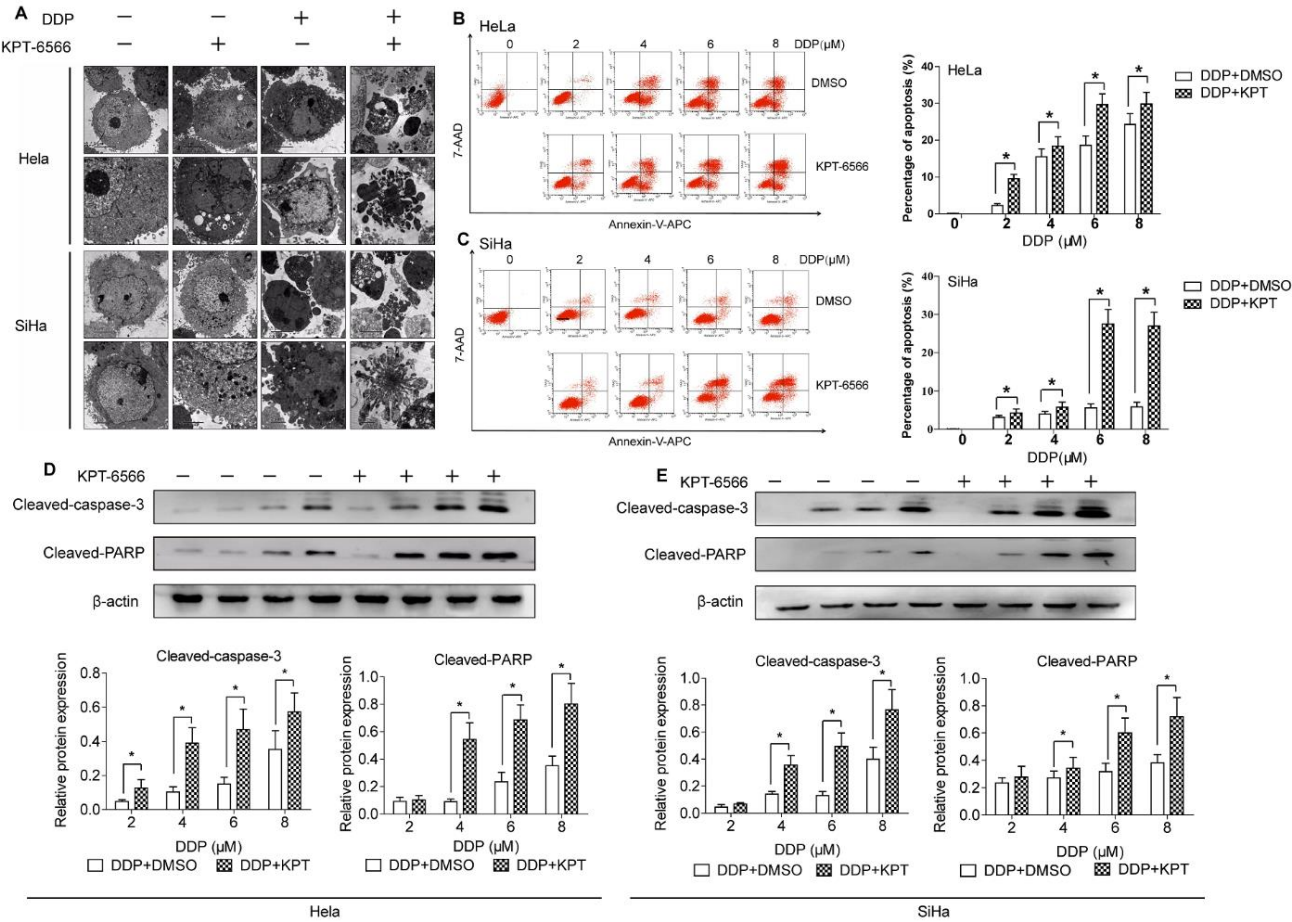
**KPT-6566 targeted approach brings better CCCs killing effects with less dose of cisplatin**. **(A)** Electron microscope findings in Hela/SiHa treated with 5 μM KPT-6566, 4 μM DDP and combation of 5 μM KPT-6566 and 4 μM DDP. (B, C) Flow cytometry to measure apoptosis of Hela/SiHa cells after treated with gradient concentration of DDP, compared with the combinational treatment. Annexin V-APC and 7-ADD positive cells were representative apoptotic cells. Each assay was performed in triplicate. *P < 0.05. (D, E) Cleaved-caspase-3, cleaved-PARP expression in Hela/SiHa treated with the same dose of DDP and KPT-6566 by using western blot assay. Each assay was performed in triplicate. * P<0.05

### KPT-6566 enhanced the lethal effects of DDP on CCCs

Since shRNA can have potential off-target effects and is difficult to deliver to tumours for cancer therapy. Therefore, we used a novel, small molecular Pin1 inhibitor, KPT-6566, that was identified through a mechanism-based screening from a compound library and shown to covalently bind to Pin1 and modify the structure of Pin1 by the addition of a sulfanyl-acetate group (-S-CH2-COOH) which induces the subsequent degradation of active Pin1. The IC50 values of DDP for HeLa and SiHa cells were 8.1 and 9.3 μM, respectively. In contrast, the IC50 values of KPT-6566 for HeLa and SiHa cells were 13.5 and 14.3 μM, respectively. After being treated with 4 μM DDP for 24 h, the cells were observed by electron microscopy. The apoptosis rate of HeLa/SiHa cells in the KPT-6566 and DDP combination treatment groups was significantly higher than that observed in the DDP group (Fig. 3A). After Pin1 knockdown, the overall cell morphology of HeLa/SiHa cells dramatically changed, with the loss of protruding pseudopodia, ribosome swelling, and mitochondrial cavitation observed (Fig. 3A). Consistent with these morphological changes, cell viability and proliferation were significantly suppressed, as discussed above. We treated the control (+DMSO) and treatment (+KPT-6566) groups with 2, 4, 6 and 8 μM DDP. The flow cytometry results showed that the KPT-6566 treatment of cells enhanced the killing effect of HeLa/SiHa cells by DDP at various concentrations (Fig. 3B, C). Supporting these results, the synergistic activation of caspase-3 and PARP was also observed under the DDP and KPT-6566 combination treatment (Fig. 3D, E). Taken together, these data indicated that KPT-6566 enhanced the killing effect of DDP on CCCs.

### KPT-6566-mediated inhibition of Pin1 blocked multiple cancer-driving pathways simultaneously in CCCs

**Figure 4.**
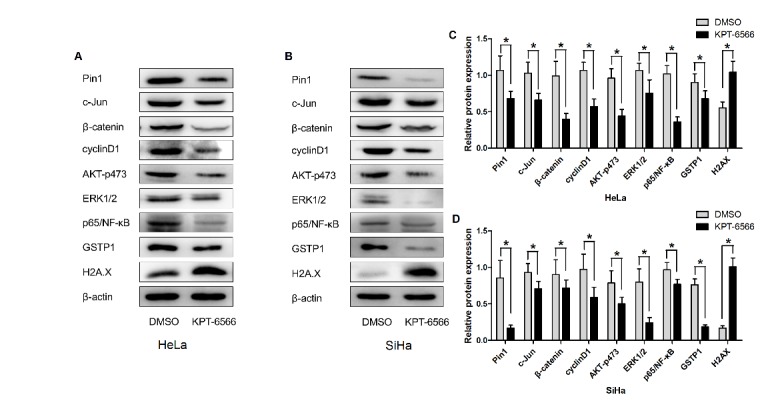
**KPT-6566 blocked multiple cancer-driving pathways simultaneously in CCCs**. **(A-D)** Hela/SiHa cells were treated with 5 μM KPT-6566. Expression of Pin1, c-Jun, β-catenin, cyclinD1, A KT-p473, ERK1/2, p65/NF-Κb, GSTP1 and H2AX were detected by western blot assay with specific antibodies. Each assay was performed in triplicate. *P<0.05.

According to the current literature, Pin1 regulates multiple cancer pathways activating more than 40 oncogenes and inhibiting more than 20 tumour suppressors. Human CCCs that were treated with KPT-6566 exhibited a significant decrease in the abundance of Pin1 and its downstream oncoproteins, including c-Jun, cyclin D1, β-catenin, ERK1/2, p-ERK, AKT, and p-AKT473 (Fig. 4A-D). Moreover, we observed that the detoxification enzyme GSTP1 was significantly decreased and that the DNA damage marker H2A.X was upregulated. These data suggest that the inhibition of Pin1 by KPT-6566 results in the suppression of multiple cancer-driving pathways. In addition, the reduction of cell detoxification and DNA damage leads to an increased chemotherapy drug sensitivity of CCCs to DDP.

**Table 3:** Correlation between Pin1 and c-Jun in SILs and SCC.

	Characteristics		No.	c-Jun		*P* value
				*Negative No.* (%)	*Positive No.* (%)	
LSIL	Pin1 status	Negative	37	19	18	0.002
		Positive	35	6	29	
HSIL	Pin1 status	Negative	4	1	3	0.747
		Positive	6	1	5	
CC	Pin1 status	Negative	10	2	8	0.049
		Positive	18	0	18	

### KPT-6566 exerted effective anticancer activity against CCCs in vivo

**Figure 5.**
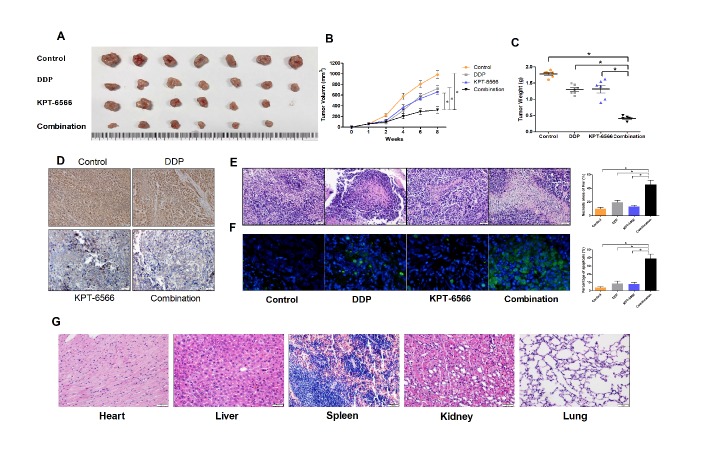
**KPT-6566 inhibited CC tumor growth by targeting Pin1 in nude mice**. **(A)** Xenograft tumors were harvested 8 weeks after implantation. **(B)** SiHa tumor volumes were measured weekly for 8 weeks and the curves of tumor volumes were plotted over time. *P < 0.05. **(C)** Data points are presented as the means ± SD for tumor weights of the tumors were analyzed. *P < 0.05. **(D)** Expression of Pin1 in xenograft tumors of nude mice were detected by IHC (original magnification × 200). **(E)** The xenograft tumor sections were subjected to H&E staining, and the percentage of the necrosis areas were counted. *P < 0.05. **(F)** The percentage of CC cell apoptosis internal xenograft tumor was counted through TUNEL. *P < 0.05. **(G)** The heart, liver, spleen, kidney and lung sections of combinational treatment nude mice were subjected to H&E staining.

To explore the potential of KPT-6566 for the clinical treatment of CCCs and to determine KPT-6566 enhances the anti-tumour efficacy of DDP in CCCs in vivo, we subcutaneously injected SiHa cells into nude mice to establish xenograft tumour models. When the tumour volumes reached 60 mm^3^, the mice were randomly grouped into four groups and treated with 20 mg/kg DDP, 5 mg/kg KPT-6566, a combination of DDP and KPT-6566 or a saline vehicle. KPT-6566 and DDP alone mildly inhibited the tumour growth in nude mice (Fig. 5B), whereas the combination of KPT-6566 with DDP significantly inhibited tumour growth. We sacrificed the nude mice using an automated CO_2_ delivery system and harvested the xenograft tumour 8 weeks after implantation. The results showed that both the weight (Fig. 5C) and volume of the tumours (Fig. 5A) were significantly decreased in the DDP and KPT-6566 combination treatment group compared with those from the other groups. We performed immunohistochemistry analyses on the xenograft tumour tissues to examine the expression of Pin1, with the results showing that KPT-6566 significantly reduced Pin1 expression in these tissues (Fig. 5D). Through H&E staining, we observed that the amount of necrotic transplanted tumour tissues in the combined treatment group was significantly higher than that observed in the other groups (Fig. 5E). We also observed significant levels of apoptosis in the xenograft tumour tissues of the combined treatment group by TUNEL staining, indicating that KPT-6566 and DDP synergistically inhibited tumour growth by inducing apoptosis (Fig. 5F). Next, we tested whether our treatment was toxic to the mouse organs. We harvested the hearts, livers, spleens, lungs and kidneys of nude mice in the combined treatment group for H&E staining, and we did not observe necrotic cells in these organs (Fig. 5G). Taken together, these data collectively demonstrate that KPT-6566 amplified the ability of DDP to induce cell death and inhibited the solid xenograft tumour growth of SiHa cells in vivo withoutorgan toxicity.

### Migration and invasion of human CCCs suppressed after Pin1 inhibition

**Figure 6.**
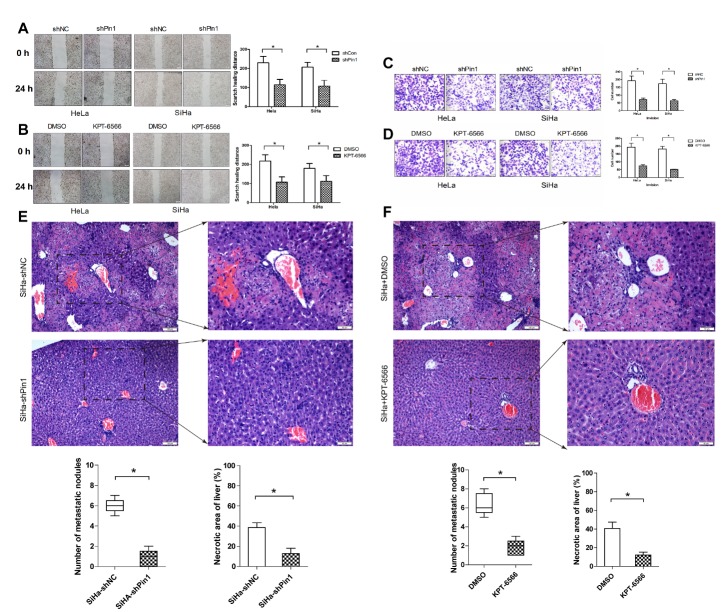
**Downregulation of Pin1 potently inhibited migration and invasion of CCCs**. **(A-D)** Representative images show the migration and invasion abilities of Hela/SiHa cells with Pin1 stably knocked down or negative control and Hela/SiHa cells treated with KPT-6566 or DMSO. The number of cells was quantified. *P < 0.05. (E, F) Hepatic metastasis model. The liver sections were subjected to H&E staining, and the metastatic nodules are indicated with arrows. The number of metastatic nodules and necrosis areas in the liver specimens were analysed (original magnification × 200). *P <0.05.

Pin1 overexpression in human LSILs is related to poor outcome and is associated with lymph node metastasis in CC patients. We investigated the morphological changes of CCCs after Pin1 knockdown by electron microscopy, and these changes may suppress CCC migration and invasion. We next investigated whether Pin1 is a key factor in these processes. The results of the wound healing assay (Fig. 6A, B) and the Matrigel invasion assay (Fig. 6C, D) showed that the inhibition of Pin1 by shRNA or KPT-6566 distinctly suppressed the migration and invasion capacity of CCCs. For the hepatic metastasis model, we placed 12 nude mice into four groups of three mice each. For groups A and B, SiHa-shNC/SiHa-shPin1 cells were injected into the spleens of the nude mice. For groups C and D, SiHa cells were injected into the spleens of nude mice, with group C subsequently receiving injections of KPT-6566 (5 mg/kg) twice a week, while group D received the same dose of DMSO as a control. After 10 weeks, the livers were collected from the mice, and the numbers of metastatic tumour nodules were examined by H&E staining. The results revealed that significantly fewer metastasis nodules were present in the Pin1-suppressed groups than in the vector group (Fig. 6E, F). We also observed that the downregulation of Pin1 reduced the liver necrosis that was caused by the CCCs (Fig. 6E, F). Therefore, we concluded that the inhibition of Pin1 by shRNA and KPT-6566 inhibited the migration and invasion of CCCs in vitro and in vivo.

### Inhibition of Pin1 suppressed the EMT of CCCs via the c-Jun/slug pathway

**Figure 7.**
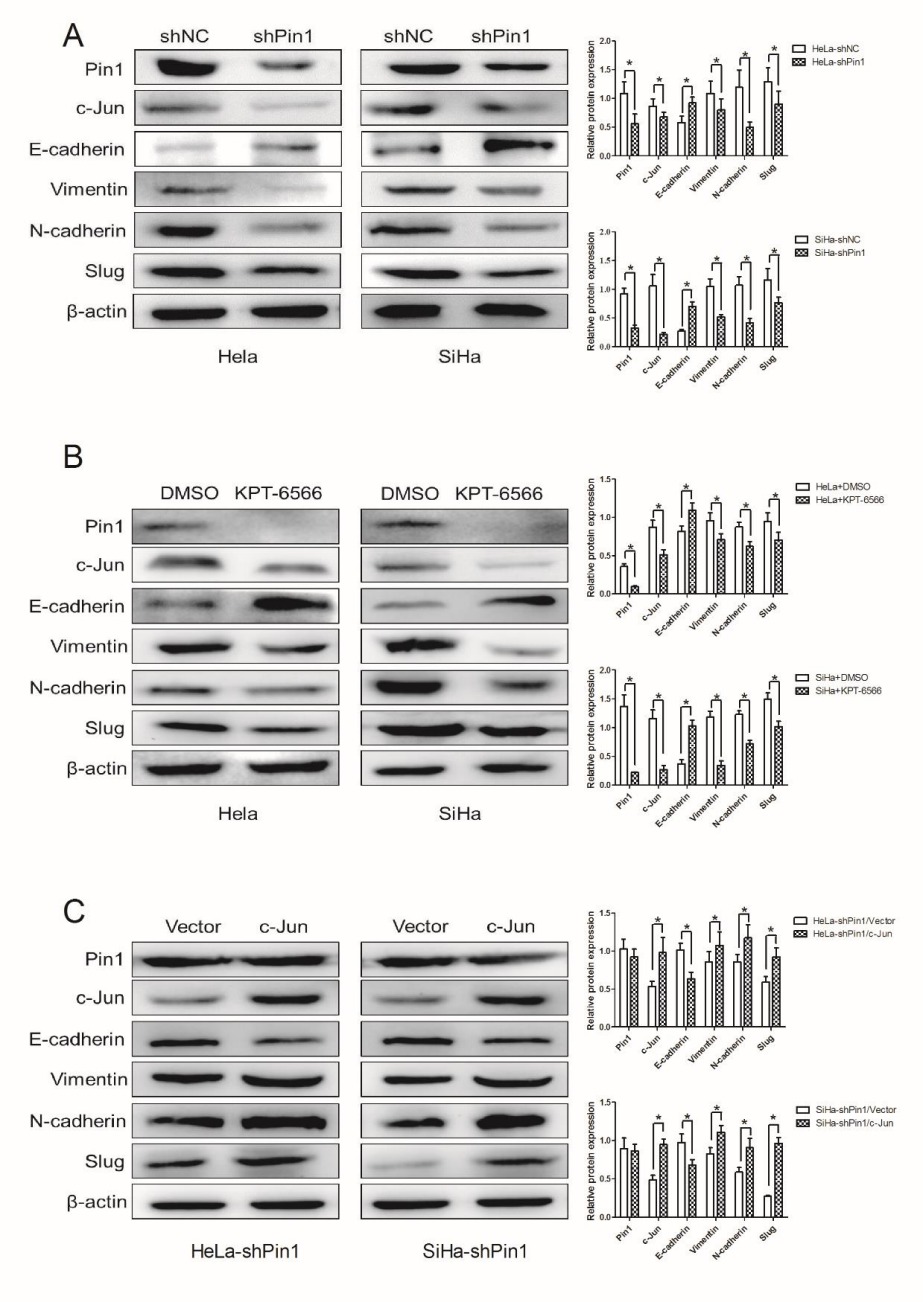
**Downregulation of Pin1 potently suppressed EMT of CCCs via c-Jun/slug pathway**. (A, B) The expression of Pin1, c-Jun, slug and EMT associated proteins including E-cadherin, N-cadherin and vimentin in Hela/SiHa cells after downregulation of Pin1 by shRNA or KPT-6566 were detected by Western blot assay. *P < 0.05. **(C)** Western blotting was performed to detect the expression of Pin1, c-Jun, slug and EMT associated proteins in Hela-shPin1/SiHa-shPin1 after c-Jun overexpression or vector. *P<0.05.

The EMT plays a crucial role in the metastasis and invasion of CCCs. Clinical data show that the EMT promotes the deep infiltration of cervical cancer cells and cervical cancer progression. We hypothesized that downregulation of Pin1 may reverse EMT in CCCs. To test this possibility, we examined the cell phenotypes of stably infected CCCs and KPT-6566-treated CCCs. Consistent with our hypothesis, protein expression of the epithelial marker E-cadherin distinctly increased in Pin1-suppressed, stable HeLa/SiHa cells, whereas expression of the mesenchymal marker’s vimentin and N-cadherin was dramatically decreased (Fig. 7A, B). Furthermore, our results revealed that c-Jun and slug were significantly downregulated in infected CCCs and in the KPT-6566-treated CCCs. The transcription factor c-Jun is a substrate of Pin1, and the regulation of slug by c-Jun plays a vital role in promoting the EMT. Overall, we observed that the inhibition of Pin1 by shRNA and KPT-6566 suppressed CCC metastasis and invasion via the c-Jun/slug pathway.

To further investigate our hypothesis, we overexpressed c-Jun in the Pin1 knockdown cell lines. We observed that expression of slug was significantly upregulated and that the EMT phenotype was restored in CCCs (Fig. 7C). Taken together, these data show that the MET phenotype induced by the inhibition of Pin1 may be associated with the c-Jun/slug pathway.

## DISCUSSION

Pin1 acts as a regulator that promotes cancer progression[20],and a comprehensive understanding of how Pin1 drives tumourigenesis is essential for cancer prevention, therapeutic,development and the rational selection of combination therapies [21, 22]. In the current study, we demonstrated that Pin1 may represent a promising target for the prevention and treatment of CC.

Pin1 participates in a variety of processes, including cell proliferation, growth suppressor evasion, genome instability and centrosome amplification, and Pin1 acts on its substrates cyclin D1, AKT, ERα, β-catenin, NF-κB, and c-MYC at the tumour initiation stage in breast cancer [8]. The conversion of LSIL to HSIL will greatly increases the risk of cervical cancer [23]. Our study first showed that LSIL patients with high expression levels of Pin1 showed significantly worse outcomes, which were associated with the ability of Pin1 to drive multiple oncogenic pathways. This result indicated that Pin1 plays a crucial role in the very early stages of CC. We observed a significantly different rate of non-progression between cancer patients with Pin1-negative and Pin1-positive statuses, and this is the first study to demonstrate a role for Pin1 IHC as a triage tool for LSIL biopsies.

Currently, the continuous development of LSIL via HSIL to cervical cancer remains to be demonstrated. Furthermore, a patient with a post-colposcopy histopathological diagnosis of LSIL still has an approximately 10% chance of harbouring an HSIL [24-27]. Therefore, it is inevitable that both LSIL and HSIL exist simultaneously. Although the majority of LSILs regress spontaneously, >10% may ultimately either progress to or have an associated HSIL [27-30]. Most cervical LSILs (80-85%) occur to due an infection with HR HPV types. In one of the larger published studies on this topic, Herfs and colleagues were the first to show a role for CK7 IHC as a triage tool for LSIL/CIN1 biopsies [31]. Subsequently, Paquette et al. [32], Mills et al. [33] and Huang et al. [34] revealed that CK7 positivity in LSIL is associated with an increased risk of developing HSIL.In our study, we showed that Pin1 may have the similar function as CK7 as a risk stratifier for LSIL. The identification of LSIL/CIN1 patients at risk for concurrent or future HSIL/CIN2-3 is of clinical interest, because at present this small subset dictates management for all LSIL/CIN1 cases. This situation leads to expensive and disruptive monitoring, particularly colonoscopies and biopsies, and potential overtreatment in a large population of women who only have LSIL.

Pin1 is involved in cancer development and progression by regulating many oncogenes, such as cyclinD1, Notch1 and mutp53 [35]. We showed that Pin1 knockdown significantly reduces the proliferation of CCCs. Moreover, we demonstrated for the first time that the novel chemical inhibitor KPT-6566 inhibits the proliferation of CCCs and significantly enhances the killing effect of cisplatin on CCCs in vitro and in vivo. Although we observed that c-Jun, cyclin D1, β-catenin, ERK1/2, p-ERK, AKT, and p-AKT473 were downregulated after KPT-6566 treatment, the underlying mechanism by which KPT-6566 is involved in regulating the chemosensitivity of CCCs remains undefined. Interestingly, we found that KPT-6566 inhibited GSTP1 expression. GSTP1 is an enzyme that exhibits diverse functions [36], including the detoxification of xenobiotic compounds, immune system evasion and apoptosis inhibition [37, 38]. It has been reported that the expression of GSTP1 is decreased after treatment with the Pin1 inhibitor ATRA in the liver cancer cell line PLC/Huh-7 [15]. However, the mechanism of KPT-6566-mediated GSTP1 downregulation is still unclear. Furthermore, p53 positively regulates the expression of GSTP1 in cancer, and Pin1 acts on p53 to regulate its stability [39-41]. However, whether Pin1 regulates GSTP1 by regulating the stability of p53 remains unclear and needs to be further studied.

Current research has indicated that blocking a single pathway is often ineffective for the treatment of solid tumors [42]. As a regulator of phosphor-proteins with the Ser/Thr-Pro motif, Pin1 regulates multiple cancer-driving pathways in CCCs [9]. Based on the use of conventional chemotherapy drugs, the effective inhibition of Pin1 will greatly improve the therapeutic effects on CC [43]. Cisplatin is one of the first-line chemotherapy drugs for the clinical treatment of cervical cancer, but in some cases, cisplatin has a limited killing effect on CCCs[44]. High doses and the long-term use of cisplatin can cause severe nephrotoxicity [45, 46]. Our study explored the possibility of combining KPT-6566 and cisplatin. Combination therapy significantly reduced the necessary dose of cisplatin and enhanced its anti-tumour effects. Importantly, our treatment did not harm the organs of nude mice. The growth of the xenograft tumours in mice that were treated with this method was reduced, and there was necrosis and apoptosis inside the xenografted tumours, indicating that our treatment could inhibit the growth of CCCs in mice and effectively kill the tumours. Cisplatin kills tumours is by causing DNA lesions [47], inhibiting cell mitosis [48] and causing mitochondrial death [49]. We observed a significant increase in the DNA damage protein H2A.X and in the amount of cellular mitochondrial cavitation after KPT-6566 treatment. These synergistic phenomena may be the mechanisms by which of KPT-6566 enhances the efficacy of cisplatin. Our results suggest suggested that Pin1 could be a potent therapeutic target in cervical cancer treatment, and we believe that KPT-6566 has good prospects for the treatment of cervical cancer in combination with anti-tumour drugs.

Although there is a very poor prognosis for patients with CC after the cancer has metastasized, the underlying mechanisms that cause the metastatic cascade remain undefined [50]. Pin1 participates in the modulation of cancer cell plasticity, shape and migratory abilities [8]. Furthermore, Pin1 overexpression was observed to downregulate E-cadherin and upregulate N-cadherin and vimentin in both normal and cancerous mammary epithelial cells to promote the EMT via the PI3K-AKT, NF-κB or Notch1 pathways [51]. The EMT is a phenotypic alteration that converts adherent epithelial-like cells into individual-like cells [52]. The EMT is correlated with distal metastasis and the depth of invasion of CCCs [53]. In our study, we showed that the suppression of Pin1 by shRNA or by the novel chemical inhibitor KPT-6566 reduced the migration and invasion abilities of CCCs. Moreover, we discovered that the downregulation of Pin1 inhibited c-Jun/slug expression and further suppressed the EMT process. Notably, the suppression of Pin1 efficiently repressed the protein expression levels of N-cadherin and vimentin and enhanced the expression level of E-cadherin. These results are consistent with those of the wound healing and transwell assays. To further elucidate the mechanism by which Pin1 promotes the EMT in CCCs, we showed that the overexpression of c-Jun in Pin1-silenced CCCs significantly restored its EMT phenotype with slug overexpression. We revealed a novel molecular mechanism by which Pin1 promoted the EMT in CCCs via the c-Jun/slug pathway. In a liver metastasis model of CC in nude mice, we demonstrated that the interference of Pin1 expression led to a decrease in the levels of distant metastasis and liver necrosis that occurred. Here, we simultaneously investigated the functions of Pin1 and its inhibitor KPT-6566 in the processes of CCC infiltration and metastasis and elucidated the associated molecular mechanisms.

Several studies have shown that Pin1 can activate multiple cancer-promoting pathways in human cancers [54]. Several Pin1 inhibitors have been identified to date, such as juglone, PiB and ATRA; these inhibitors have both covalent and non-covalent mechanisms of action [19]. Low-throughput PPIase screens have identified juglone, which can covalently modify the active site of Pin1, although this compound has several off-target effects and exhibits Pin1-independent activities [55]. PiB exerts its effect through Parvulin 14 inhibition and demonstrates a low specificity for Pin1 [56]. ATRA promotes Pin1 degradation with a non-covalent mechanism, but its half-life is very short [12]. These issues have become obstacles for the development of new anticancer drugs using ATRA as a precursor. KPT-6566 is highly specific towards Pin1 and primarily inhibits the expression of Pin1 in CCCs, through a mechanism that is similar to that of shRNA. Above all, we demonstrated in animal experiments that KPT-6566 exerts anticancer effects and is not life-threatening.

Taken together, we have shown that Pin1-positive LSILshave a higher risk for future HSIL compared with a Pin1-negative LSILs. The expression of Pin1 was positively associated with lymph node metastasis and c-Jun expression in human cervical cancer tissues. The genetic and KPT-6566 inhibition of Pin1 inhibited CCC proliferation, migration and invasion in vitro and in vivo. The KPT-6566 inhibition of Pin1 blocked multiple cancer-driving pathways simultaneously and enhanced the lethal effects of DDP on CCCs in vitro and in vivo. In summary, we showed that Pin1 may be a marker for the risk of progression to HSIL, and KPT-6566 may be further developed as a cancer therapeutic that inhibits Pin1.

## Supplementary Materials


