# Supplementary material for: Predictive Value of Pin1 in Cervical Low-Grade Squamous Intraepithelial Lesions and Inhibition of Pin1 Exerts Potent Anticancer Activity against Human Cervical Cancer
Source: Aging Dis. 2020 Feb 1;11(1):44–59. doi: 10.14336/AD.2019.0415 (PMC6961766; doi:10.14336/AD.2019.0415)
Supplement: Supplementary file 1 [file AD-11-1-44-s.pdf]

SUPPLEMENTARY DATA

Predictive Value of Pin1 in Cervical Low-Grade Squamous Intraepithelial Lesions and Inhibition of Pin1 Exerts Potent Anticancer Activity against Human Cervical Cancer

Yan-Tong Guo, Yan Lu, Yi-Yang Jia, Hui-Nan Qu, Da Qi, Xin-Qi Wang, Pei-Ye Song, Xiang-Shu Jin, Wen-Hong Xu, Yuan Dong, Ying-Ying Liang, Cheng-Shi Quan*

Key Laboratory of Pathobiology, Ministry of Education, College of Basic Medical Sciences, Jilin University, Changchun, China.

© 2019. Guo YT et al. Published online at http://www.aginganddisease.org/EN/10.14336/AD.2019.0415

SUPPLEMENTARY DATA

Supplementary Table 1. P16 and Ki67 expression according to Pin1 statuses in SILs.

Characteristics		No.	LSIL		*P* value	No.	HSIL		*P* value
			Pin1 Negative No. (%)	*Pin1 Positive No.*(%)			*Pin1 Negative No.* (%)	*Pin1 Positive No.*(%)	
	No.		37	35			4	6	
p16	Negative	23	14	9	0.270	1	1	0	0.197
	Positive	49	23	26		9	3	6	
Ki67	1%-25%	11	10	1	**0.010**	0	0	0	**0.010**
	25%-50%	10	7	3		0	0	0	
	50%-75%	23	9	14		5	4	1	
	>75%	28	11	17		5	0	5	

Supplementary Table 2. Clinical trials of SIL patients.

Characteristics		*LSIL*	*HSIL*	*P* value
	No.	72	10	
Age (years)	<50	43	3	0.076
	≥50	29	7	
Colposcopic diagnosis	Normal/benign	21	0	**<0.001**
	LSIL	47	2	
	HSIL	4	8	
	MIC/invasive cancer	0	0	
Transformation zones	type I	28	1	**0.034**
	type II	23	2	
	type III	21	7	

© 2019. Guo YT et al. Published online at http://www.aginganddisease.org/EN/10.14336/AD.2019.0415

SUPPLEMENTARY DATA

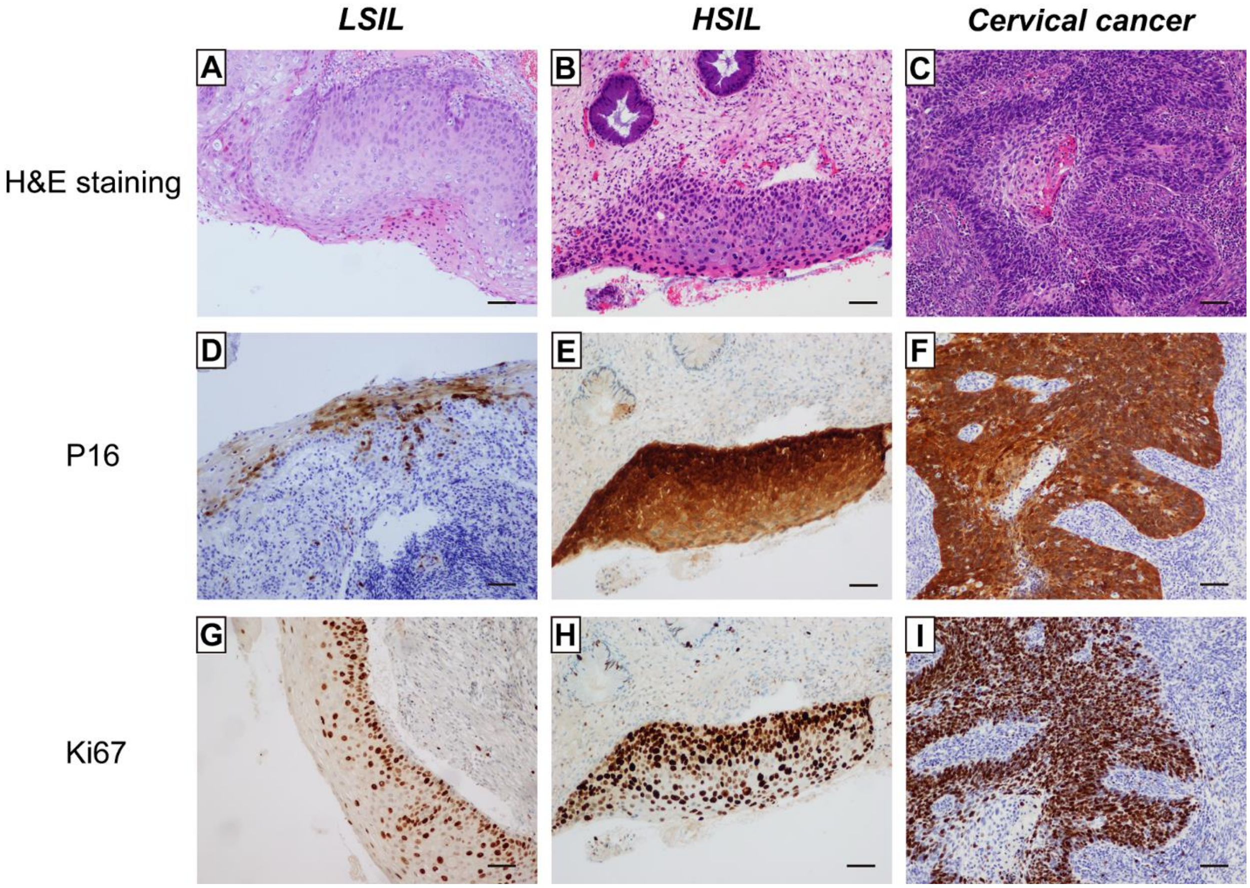

Supplement Figure 1. H&E stain and IHC findings in LSIL, HSIL and SCC. (A-I) Representative images of hematoxylin and eosin (HE) staining and p16 & Ki67 immunostaining of LSIL, HSIL and cervical cancer tissues. Scale bar = 50 μm.

© 2019. Guo YT et al. Published online at http://www.aginganddisease.org/EN/10.14336/AD.2019.0415
